# The effect of age and sex on selected hematologic and serum biochemical analytes in 4,804 elite endurance-trained sled dogs participating in the Iditarod Trail Sled Dog Race pre-race examination program

**DOI:** 10.1371/journal.pone.0237706

**Published:** 2020-08-20

**Authors:** Sara L. Connolly, Stuart Nelson, Tabitha Jones, Julia Kahn, Peter D. Constable

**Affiliations:** 1 Department of Veterinary Clinical Medicine, College of Veterinary Medicine, University of Illinois, Urbana-Champaign, Illinois, United States of America; 2 Iditarod Trail Committee, Wasilla, Alaska, United States of America; 3 Veterinary Specialty Center, Buffalo Grove, Illinois, United States of America; Animal Health Centre, CANADA

## Abstract

Endurance-trained sled dogs provide a unique translational model to characterize changes in hematologic and serum biochemical analytes due to the aging process. The primary objective of this study was to determine the effect of age and sex on specific hematologic and serum biochemical parameters in the endurance trained sled dog. Longitudinal and cross-sectional data were analyzed from 9,746 blood and serum samples from 4,804 dogs collected over 7 years as part of the Iditarod Trail Sled Dog Race pre-race examination program. Mixed models analysis was used for statistical analysis and P < 0.01 was considered significant. Dogs ranged from 1–12 years of age and 39% were female. Serum total calcium and phosphorus concentrations and white blood cell count decreased nonlinearly to asymptotic values by 6.6, 3.1, and 6.9 years of age, respectively, equivalent to estimated physiologic ages in human years of 44, 27, and 46 years. Serum glucose concentrations reached their lowest value at 7.8 years of age, equivalent to an estimated human physiologic age of 50 years, after which time the concentration increased. Serum globulin concentrations increased with age, but nonlinearly for females and linearly for males. Most sex-related differences were <5%; however, females had lower serum urea nitrogen (14.7%) and creatinine (7.3%) concentrations, lower serum alanine aminotransferase activity (16.6%), and higher serum total bilirubin concentration (12.8%) and platelet count (6.0%). The endurance-trained sled dog provides an excellent model to separate the physiologic effects of age from those of a sedentary lifestyle on hematologic and serum biochemical analytes.

## Introduction

Sustained physical activity throughout the human life span appears to be the evolutionary norm; however, most inhabitants of developed countries have adopted a sedentary lifestyle that distorts investigations of the aging process. Much of the data being analyzed for human aging studies therefore represents the aging process superimposed on the pathophysiologic consequences of inactivity [1]. Studies of the aging process in physically active subjects are therefore of great interest. The endurance-trained sled dog is unique in that this population incorporates a highly trained group of mixed breed female and male dogs with a widely varying age but similar incidence of age-related diseases as humans [2–4]. A sled dog is any dog trained to pull a sled with a musher in the context of a team. These dogs should demonstrate a willingness to be harnessed and a desire to participate in the activity for which it is harnessed. Endurance trained sled dogs are trained to run long distances ranging from 150 to 1,000 miles over a length of days. Although mixed breed in nature, some degree of purposeful breeding does occur to foster specific athletic qualities. Sled dogs also provide a naturally occurring comparative model to humans for the physiological effects of endurance exercise, including plasma volume expansion and development of the athlete’s heart [5]. It is often overlooked that humans evolved as elite endurance runners, defined as running many kilometers over an extended period using aerobic metabolism [6]. A capacity for endurance running is not evident in other primates and is uncommon in quadrupedal mammals other than dogs and hyenas and migratory ungulates such as wildebeest and horses [6]. Studies of endurance-trained sled dogs provide an opportunity to accurately characterize age-related changes in hematologic and serum biochemical analytes that are not influenced by the physiologic effects of an inactive lifestyle. Our first hypothesis was therefore that endurance-trained sled dogs have age related changes in hematology and serum biochemistry that differ from those previously noted in less active companion dogs and sedentary humans.

Establishment of species-specific reference intervals allows the clinician to identify organ systems and physiologic derangements that are worthy of further investigation. Reference intervals can be influenced by physiologic differences including age, sex and breed [7–12] making establishment of reference intervals for niche populations of clinical relevance. Our second hypothesis was that endurance-trained sled dogs have unique hematologic and serum biochemistry reference intervals compared to the companion dog. The primary aims of this study were therefore to characterize the hematologic and serum biochemical changes that occur with age and between the sexes in the endurance-trained sled dog, and to establish appropriate hematologic and serum biochemistry reference intervals for this population. In order to accomplish these aims, we analyzed a large data set obtained as part of the pre-race examination of dogs running in the Iditarod Trail Sled Dog Race over a seven-year period.

## Material and methods

Blood samples were collected as part of the standard pre-race examination of all dogs being considered for participation in the Iditarod Trail Sled Dog Race over seven consecutive years (2012 to 2018). The Iditarod Trail Sled Dog Race is run annually in early March in Alaska from Anchorage to Nome and is widely regarded as the premier long-distance sled dog race in the world. Dogs completing this race typically run 100 miles each day for 10 consecutive days while pulling a sled with a musher. The extraordinarily high daily mass specific energy expenditure (4,400 kJ.kg^-0.75^) required to accomplish this feat is the highest recorded for any vertebrate and more than three times that of cyclists competing in the Tour de France [13].

Each musher was permitted to present up to 24 endurance-trained dogs for testing. The samples were collected for diagnostic purposes and not as a part of this study. The authors were given permission to use the data after analysis for diagnostic purposes had taken place. As this testing is a requirement of race participation and a veterinary-client-patient relationship was present at the time of collection, ethical approval from our institution was not required [14, 15]. Dogs were rested from exercise for at least 12 hours and fed and watered normally before blood collection. Signalment data acquired from the musher or team handler included age, sex, and name of the dog. Information regarding the neuter status of the dog was not available. Based on the long-standing involvement of one of the authors with the race (SN) it is estimated that approximately 50% of the dogs (males and females) are neutered. A microchip identification number was also recorded for all dogs and used as the unique identifier for data analysis. Sample collection occurred at up to four sites in Alaska within one month of the beginning of the race, typically during the entire month of February, and samples were processed at Providence Alaska Medical Center (Anchorage, AK), a human laboratory accredited by the College of American Pathologists and the Clinical Laboratory Improvement Amendments. Data from the years 2012 to 2018 were retrieved for analysis because the same analyzers were used to process the samples during the seven-year study period.

Jugular venous blood samples were collected with the dogs manually restrained in a sitting position by a registered veterinary technician using a 21-G x 1-1/4” BD Vacutainer Eclipse blood collection needle (Becton, Dickinson and Company, Franklin Lakes, NJ). Blood collection was supervised by the same head veterinary technician during all years and samples were always collected indoors. The veterinary technicians involved in blood collection varied from year to year. Blood was collected into a 3ml K^+^-EDTA BD Vacutainer tube for hematology and a 5ml BD Vacutainer serum separator tube with silica clot activator, polymer gel and silicone-coated interior for serum biochemistry. The K^+^-EDTA samples were mixed by manual inversion directly after collection to prevent clotting. Whole blood samples for serum analysis were allowed to clot for 30–60 minutes at approximately 10 to 20 °C and then centrifuged at 3100 x g for 10 minutes. As an additional part of the pre-race examination, a six-lead electrocardiogram (ECG) was obtained on each dog by veterinary technicians after blood sampling. The dogs were restrained in right lateral recumbency and the ECG recorded and analyzed as reported elsewhere [16]. The ECG records included the microchip identification number and signalment of each dog.

Blood samples were stored in a non-heated room with a temperature of approximately 4 to 10 °C for up to 10 hours before being transported and processed within 24 hours using the Beckman Coulter DxH 800 (Beckman Coulter, Brea, CA) for hematologic analysis and the Beckman Coulter AU680 (Beckman Coulter, Brea, CA) for serum biochemical analysis. Hematologic analytes measured included total white blood cell (WBC) count, blood hemoglobin (Hgb) concentration, hematocrit (Hct), mean corpuscular volume (MCV) and platelet (Plt) count. Evaluation of a blood smear was not performed. Serum biochemical analytes measured included the concentrations of sodium (Na, indirect ion selective potentiometry (IISP)), potassium (K, IISP), chloride (Cl, IISP) glucose (Gluc, hexokinase) total protein (TP, biuret), albumin (Alb, bromocresol green), globulins (Glob, calculated as the difference between TP and Alb concentrations), total calcium (Ca, arsenazo III), phosphorus (Phos, heteropolyacid complex), serum urea nitrogen (SUN, NADH method), creatinine (Creat, modified Jaffe procedure), and total bilirubin (Tbili, modified diazo method), as well as the activity of alkaline phosphatase (ALP, p-nitro-phenylphosphate method), creatine kinase (CK, NADPH method), aspartate aminotransferase (AST, modified IFCC method) and alanine aminotransferase (ALT, modified IFCC method).

Data were compiled in a Microsoft Excel spreadsheet by two of the authors, SC and JK, with each year of data organized as a separate worksheet. Once all data were assembled, the entire dataset was sorted by microchip ID and year to allow for evaluation of the chronologic order of age, possible duplication of data entries and inconsistencies in recording sex. Entries that raised concern regarding pre-analytical transcription errors were confirmed or refuted by repeat evaluation of the microchip ID and signalment information available with the clinical pathology results as well as the ECG results. Data from a later entry were removed when duplicate entries were identified within a single year based on the microchip identification number. Appropriate age chronology was verified by evaluating all years of data available for each unique microchip identification number.

### Statistical analysis

Statistical analysis was performed using SAS version 9.4 (SAS Institute Inc., Cary, NC). Statistical significance was set at *P* ≤ 0.01 because of the large number of dogs in the study population and our interest in identifying factors of clinical or physiologic importance [17]. Spearman’s rho (r_s_) and Pearson’s correlation coefficient (r) were calculated to characterize the curvilinear and linear association, respectively, between analytes and age using data from the last race for each dog. Data was considered curvilinear if the best fit line was described by a quadratic equation (*P* < 0.01 for x^2^ coefficient) and linear if the best fit line was described by a slope-intercept line equation (*P* ≥ 0.01 for x^2^ coefficient when a quadratic equation was applied). Spearman’s rho correlates the rank value between two variables and is therefore used for curvilinearly-related data whereas Pearson’s correlation coefficient is used for linearly-related data.

The study contained both longitudinal (repeated data from the same subject over time) and cross-sectional (data from multiple subjects at a single time point) data and therefore was analyzed using mixed models methods (PROC MIXED). Mixed models analysis permits the simultaneous evaluation of longitudinal and cross-sectional data as well as fixed (age, sex, sampling year) and random (team) effects.

The mixed models procedure was applied to the complete data set using a repeated statement and dog nested within a musher’s team to account for potential confounding effects of nutritional, managemental and environmental factors. Sampling year was included to account for potential year to year variation in analyzer performances. A compound symmetry structure was selected for mixed models analysis based on the restricted maximum likelihood (REML) to estimate variance and covariance components. The Kenward-Roger approximation for the model-based REML was used to estimate the covariance within the model as this method accounts for missing covariates when covariates are missing at random. In this study dogs were clustered within team, such that Y*ij* denotes dog *i* in team *j*; application of the compound symmetry structure assumes that all dogs in each team change in the same way over sampling year, such that the total variation in Y*ij* can be partitioned into the variation within a team, σ^2^_ε_, and the variation between teams, σ^2^_γ_.

Main effects initially considered in the mixed models procedure were age in years (continuous; 11 levels, with dogs aged 11 and 12 being collapsed into one category identified as 11.5 years because of the sparseness of data), sex (categoric; 2 levels, female, male), the interaction between age and sex, and sampling year (categoric; 7 levels). Data points with a studentized residual >3.5 were identified and considered as outliers in the mixed models analysis if they were considered physiologically unlikely by the authors in an endurance-trained athlete, in which case they were treated as missing data for statistical analysis. The involvement of two of the authors (SN, PDC) with the Iditarod Trail Sled Dog Race spans more than 20 years providing them appropriate knowledge regarding what is physiologically possible within this population. Inclusion of these data points did not change any conclusions of the statistical analysis but produced slightly wider 95% confidence intervals for the estimates of significant predictors. Non-significant interaction effects between age and sex were deleted from the model when summarizing the final mixed model for each analyte.

Three mixed models were compared to best characterize the relationship between the analyte and age when a significant main effect of age was identified. The three models were a linear equation (PROC MIXED), a curvilinear (quadratic) equation (PROC MIXED), and a nonlinear equation (PROC NLMIXED). The model with the lowest value for Akaike’s Information Criterion was selected as the model equation for that analyte as the model with the lowest Akaike’s Information Criterion value indicated the best fit to the data over the entire age range. The nonlinear equation was an exponential decay or increase to a non-zero asymptote (A_sym_; y = A_sym_ + (A_0_-Asym)×*e*^(-b×Age)^), depending on whether the analyte of interest decreased or increased with age. Separate analyses for sex (female or male) were conducted for the nonlinear equation when significant effects of sex, and the interaction between sex and age, were identified. A dummy variable for female (female = 1; male = 0) was added to the nonlinear equation when a significant effect of sex was identified and the interaction between sex and age was not significant. For analytes best modeled by a nonlinear equation, the age was calculated at which the predicted mean value first decreased to the upper value of the 95% confidence interval for the asymptote estimate.

To assist comparison to aging in humans, the physiologic ages of dogs in human years were calculated as described for mixed breed dogs weighing between 23 and 34 kg [18]. This weight range included most dogs in the study population, based on reported mean ± SD body weights for endurance-trained sled dogs running in the Iditarod Trail Sled Dog Race of 26.7 ± 3.9 kg (n = 319) [19] and 23.9 ± 4.0 kg (n = 100) [20]. The age of dogs in years in the study population, with the estimated equivalent physiologic age in human years in parentheses, were: 1 (15), 2 (21), 3 (27), 4 (32), 5 (37), 6 (42), 7 (46), 8 (51), 9 (55), 10 (59), and 11.5 (65). These comparative estimates of age should be considered shorter than the true value because they were derived from a population of dogs that were sick and referred to tertiary care veterinary teaching hospitals for diagnosis and treatment [21]. Nevertheless, the consensus view is that dogs > 22.7 kg body weight should be classed as senior when 6 to 8 years-of-age and geriatric when they are ≥ 9 years-of-age [3]. Although comparison of canine to human age is not perfect and medical practices differ between species which may affect age status, more specifically in the dog, we feel there is potential for using the dog as a comparative model in human aging research.

Reference intervals were determined using MedCalc statistical software version 19.0.5 (MedCalc Software bvba, Ostend, Belgium) and a non-parametric percentile method as recommended by the Clinical and Laboratory Standards Institute guideline C28-A3 [22]. The reference intervals were determined by including all data points, even those identified as outliers by mixed models analysis since all outliers were thought to be physiologically possible within this population. Data points with a studentized residual > 3.5 (equivalent to *P* < 0.00023 for a Z score) were excluded from mixed models analysis. It should be noted that these values have no influence on the 95% reference interval when the interval is calculated using the non-parametric percentile method, as these data points lie well outside the range that contains 95% of all values.

## Results

The final data set consisted of 9,746 data points from 4,804 dogs in 195 teams during the seven-year study period. The number of data points from female and male dogs in the final data set were 3,766 (38.6%) and 5,980 (61.4%), respectively. A small proportion of females (number not tracked) may have been undergoing a heat cycle during blood sampling as these females were not excluded from participation in the race.

Dogs ranged from one to twelve years of age, being aged one year (655 data points), two years (1,710), three years (1,861), four years (1,683), five years (1,397), six years (1,039), seven years (724), eight years (421), nine years (185), ten years (61), and eleven and twelve years (10). The number of dogs sampled varied from year to year, with year 2012 (1,158), 2013 (1,327), 2014 (1,386), 2015 (1,580), 2016 (1,667), 2017 (1,333), and 2018 (1,295). Dogs ran in 1 race (n = 2,399, 49.9%), 2 races (n = 1,066, 22.2%), 3 races (n = 615, 12.8%), 4 races (n = 386, 8.0%), 5 races (n = 227, 4.7%), 6 races (n = 86, n = 1.8%) or in all 7 races (n = 25, 0.5%).

Heart rate was calculated from the 10 second ECG recording for 2,177 individual dogs in the study. Heart rate was similar for male and female dogs (P = 0.10; overall mean, 102 beats/min), but was weakly and negatively associated with age (r = -0.11, P < 0.0001).

Spearman and Pearson correlation coefficients for the relationship between selected analytes and age were calculated from the 4,804 dogs, comprising 1,868 (38.9%) females and 2,936 (61.1%) males (Table 1). Analytes with r_s_ ≥ 0.10 for age were serum total Ca concentration (r_s_ = -0.28), serum glucose concentration (r_s_ = -0.22), white blood cell count (r_s_ = -0.20), and serum globulin concentration (r_s_ = 0.10). Ten other analytes were significantly associated with age, but all had a r_s_ value that was < 0.10. Table 1 also provides the associated *P* values for each analyte regarding age, sex, the age/sex interaction and sampling year.

**Table 1:** Summary of the main effects of age, sex, and year of sampling, and the interaction between age and sex for selected hematologic and serum biochemical analytes.

				Mixed models analysis							
				Main effects of Age			Main effects of Sex				
Analyte	n	Spearman Correlation Coefficient for Age r_s_, (P value)	Pearson Correlation Coefficient for Age r	Linear Age Intercept (SE)	Linear Age coefficient (SE)	Age	Female (SE)	Male (SE)	Sex	Age*Sex	Year of Sampling
						P value			P value	P value	P value
**Age, Sex, Age & Sex Interaction, and Year of Sampling significant**											
Globulin (g/L)	9737	0.10 (<0.0001)	0.09	NA^¶^	Nonlinear^¶^	<0.0001	28.15 (0.06)	29.27 (0.05)	<0.0001	<0.0001	<0.0001
Total protein (g/L)	9746	0.03 (0.017)	0.03	NA^§^	Nonlinear^§^	<0.0001	60.58 (0.07)	61.39 (0.05)	<0.0001	0.0031	<0.0001
**Age, Sex, and Year of Sampling significant**											
Glucose (mmol/L)	9744	-0.22 (<0.0001)	-0.21	NA	Quadratic	<0.0001	5.50 (0.01)	5.44 (0.01)	<0.0001	0.73	<0.0001
Albumin (g/L)	9737	-0.07 (<0.0001)	-0.08	32.51 (0.08)	-0.091 (0.012)	<0.0001	32.42 (0.004)	32.11 (0.003)	<0.0001	0.084	<0.0001
Platelets (x10^9^/L)	9736	0.06 (<0.0001)	0.07	307.3 (3.2)	3.7 (0.5)	<0.0001	314.4 (1.6)	296.7 (1.3)	<0.0001	0.20	<0.0001
Hematocrit (L/L)	9738	-0.06 (<0.0001)	-0.05	0.483 (0.001)	-0.0008 (0.0002)	<0.0001	0.486 (0.001)	0.483 (0.001)	0.0005	0.025	<0.0001
Hemoglobin (g/L)	9734	-0.05 (0.0010)	-0.05	163.0 (0.4)	-0.20 (0.06)	0.0010	162.9 (0.2)	161.5 (0.2)	<0.0001	0.024	<0.0001
log_10_(Alkaline phosphatase) (U/L)	9744	-0.05 (0.0017)	-0.03	1.554 (0.007)	-0.0074 (0.0009)	<0.0001	1.548 (0.004)	1.569 (0.003)	<0.0001	0.013	<0.0001
Total bilirubin (μmol/L)	9746	-0.03 (0.034)	-0.02	2.63 (0.03)	-0.014 (0.005)	0.0027	2.86 (0.02)	2.53 (0.01)	<0.0001	0.33	<0.0001
**Age and Year of Sampling significant**											
Calcium (mmol/L)	9746	-0.28 (<0.0001)	-0.26	NA	Nonlinear	<0.0001	NA	NA	0.79	ND	<0.0001
log_10_(White blood cells) (x10^9^/L)	9740	-0.20 (<0.0001)	-0.20	NA	Nonlinear	<0.0001	NA	NA	0.023	ND	<0.0001
Phosphorus (mmol/L)	9745	-0.09 (<0.0001)	-0.09	NA	Nonlinear	<0.0001	NA	NA	0.083	ND	<0.0001
Chloride (mmol/L)	9746	0.09 (<0.0001)	0.07	111.50 (0.08)	0.065 (0.012)	<0.0001	NA	NA	0.65	ND	<0.0001
Mean corpuscular volume (fL)	9736	0.07 (<0.0001)	0.07	69.32 (0.08)	0.040 (0.011)	0.0005	NA	NA	0.15	ND	<0.0001
Potassium (mmol/L)	9746	0.03 (0.022)	0.03	4.50 (0.01)	0.0072 (0.0017)	<0.0001	NA	NA	0.83	ND	<0.0001
**Sex and Year of Sampling significant**											
log_10_(Urea nitrogen) (mmol/L)	9745	0.01 (0.31)	0.02	NA	NA	0.73	0.832 (0.003)	0.862 (0.002)	<0.0001	ND	<0.0001
log_10_(Creatinine) (μmol/L)	9746	0.04 (0.0061)	0.04	NA	NA	0.14	1.732 (0.002)	1.764 (0.001)	<0.0001	ND	<0.0001
log_10_(Alanine aminotransferase) (U/L)	9744	0.00 (0.74)	0.01	NA	NA	0.014	1.660 (0.004)	1.739 (0.003)	<0.0001	ND	<0.0001
log_10_(Aspartate aminotransferase) (U/L)	9743	-0.05 (0.0015)	-0.03	NA	NA	0.016	1.431 (0.003)	1.450 (0.002)	<0.0001	ND	<0.0001
Sodium (mmol/L)	9732	0.00 (0.74)	-0.01	NA	NA	0.81	145.55 (0.03)	145.44 (0.02)	0.0020	ND	<0.0001
**Year of Sampling significant**											
log_10_(Urea nitrogen/Creatinine)	9745	-0.032 (0.026)	-0.01	NA	NA	0.72	NA	NA	0.49	ND	<0.0001
log_10_(Creatine kinase) (U/L)	9746	-0.06 (<0.0001)	-0.04	NA	NA	0.031	NA	NA	0.53	ND	<0.0001

The results summarize 9,746 samples from dogs (n = 4,804) being considered for running in the Iditarod Trail Sled Dog Race during a seven-year study period. The majority of dogs ran in 2 or more races. Spearman correlation coefficients for the relationship between analytes and age were determined from the last sample obtained for each dog.

^¶^Nonlinear increase with age for female but linear increase for male dogs.

^§^Nonlinear increase with age for female but constant for male dogs.

Abbreviations: n = number of samples analyzed; NA = not applicable; ND = not determined.

### Age related changes in hematology and serum biochemistry

#### Hematology

**Fig 1:**
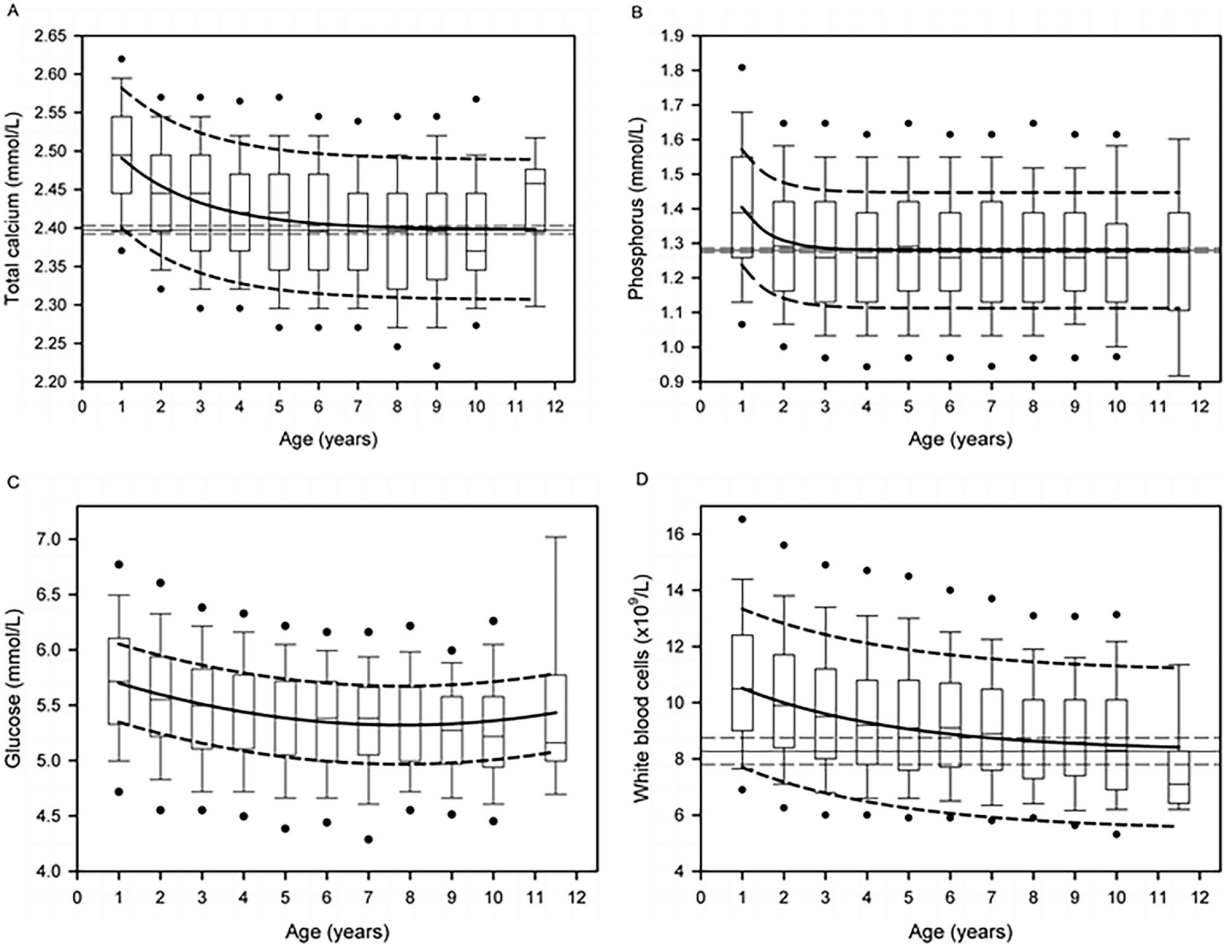
Box plots of the change in selected serum biochemical analytes (A, Calcium concentration; B, Phosphorus concentration; C, Glucose concentration) and WBC count (D) with age in 9,746 blood and serum samples from 4,804 dogs being considered for running in the Iditarod Trail Sled Dog Race. Dogs ran in 195 different teams during the seven-year study period. The box plot represents the first quartile, median, and third quartile, and the whiskers represent the 90 percentile limits. The filled circles represent the 5^th^ and 95^th^ percentile limits. The thick solid line is the regression line adjusted for repeated measures on dog as well as the effect of musher. The thick dashed lines represent the 95% prediction interval for the regression line. The thin solid line and thin dashed lines for serum Calcium and Phosphorus concentrations and WBC count represent the asymptotic value and its 95% confidence interval, respectively.

A significant effect of age and sex, but not an interaction effect, was present for hematocrit and blood hemoglobin concentration (Table 1). Both Hct and blood Hgb concentration decreased linearly with age. Age-, but not sex-, related changes were noted for the hematologic parameters WBC, MCV and Plt (Table 1). White blood cell counts decreased nonlinearly with age (r_s_ = -0.20), such that: WBC = 8.27 + 2.91×*e*^(-0.26×Age)^ (Fig 1D, Table 1). The WBC count reached the upper value for the 95% confidence interval (7.80 to 8.75 x10^9^cells/L) for the asymptote (8.27 x10^9^cells/L) at 6.9 years of age. Erythrocyte MCV increased linearly with age by 0.04 fL per year. Blood Plt counts increased linearly with age by 3.7 x10^9^/L per year.

#### Serum biochemistry

**Fig 2:**
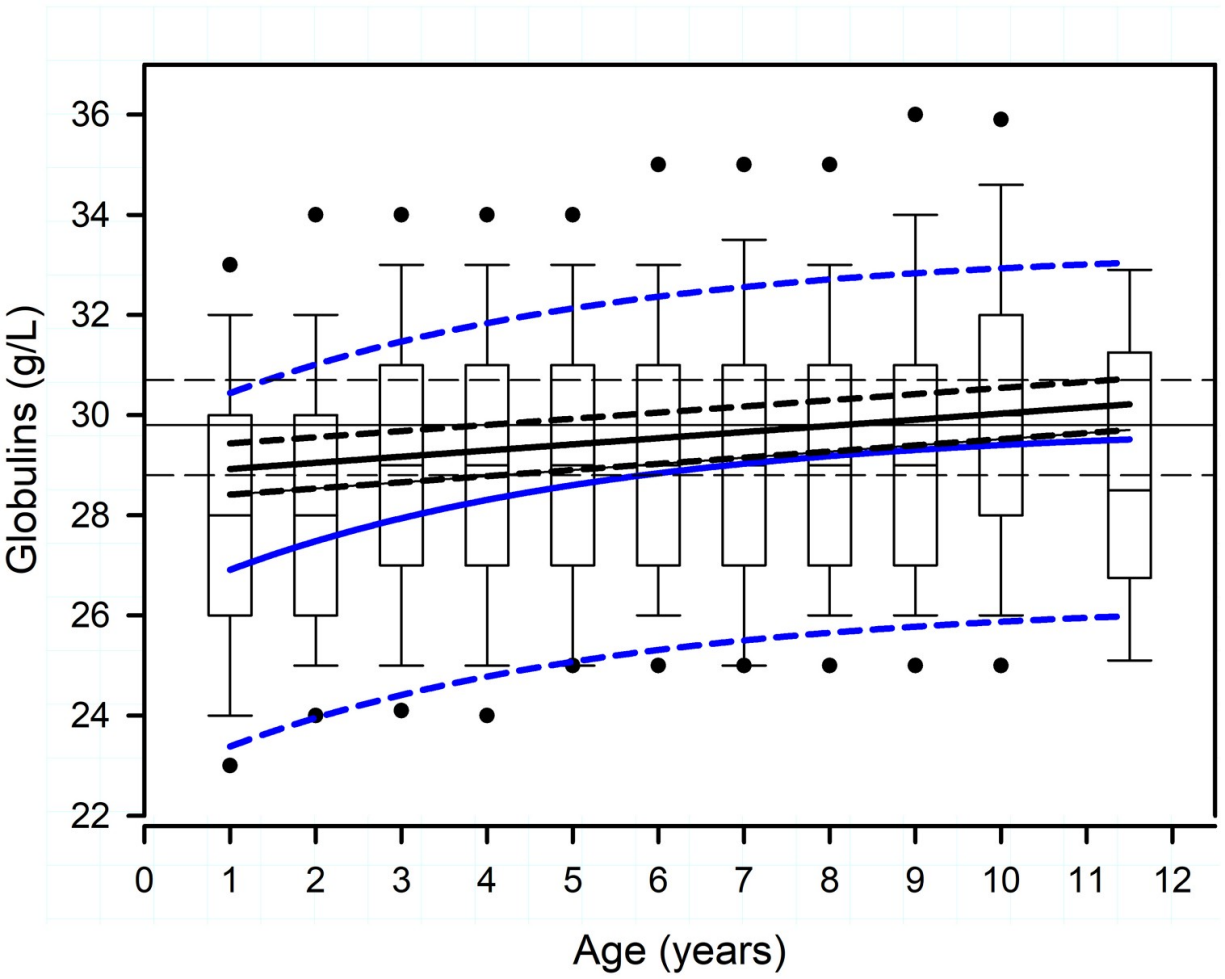
Box plots of the change in serum globulin concentration in female (n = 1,866) and male (n = 2,932) dogs being considered for running in the Iditarod Trail Sled Dog Race in 195 teams during a seven-year study period. There are 9,737 data points from 4,798 dogs. The box plot represents the first quartile, median, and third quartile, and the whiskers represent the 90 percentile limits. The filled circles represent the 5^th^ and 95^th^ percentile limits. The solid line is the regression line adjusted for repeated measures for female (blue line) and male (black line) dogs, as well as the effect of musher and year of sampling. The dashed lines represent the 95% prediction interval for the female and male regression lines. The thin solid line and thin dashed lines represent the asymptotic value for females and its 95% confidence interval, respectively.

A significant effect of age, sex, and an interaction between age and sex was present for serum globulin and total protein concentration (Fig 2, Table 1). Serum Glob concentration increased nonlinearly with age for females, such that: Glob = 29.8–3.6×*e*^(-0.22×Age)^. The serum Glob concentration reached the lower value for the 95% confidence interval (28.8 to 30.7 g/L) for the asymptote (29.8 g/L) at 5.8 years of age. In contrast, serum Glob concentration increased linearly with age for males, such that: Glob = 28.8 + 0.123×Age. Serum TP concentration increased nonlinearly with age for females, such that: TP = 61.0–2.3×*e*^(-0.56×Age)^. The serum TP concentration reached the lower value for the 95% confidence interval (60.7 to 61.3 g/L) for the asymptote (61.0 g/L) for females at 3.5 years of age. In contrast, serum TP concentration did not change with age for males (P = 0.27), such that: TP = 61.5 g/L.

A significant effect of age and sex, but not an interaction effect, was present for serum glucose, albumin, and total bilirubin concentrations and serum alkaline phosphatase activity (Table 1). Serum Gluc concentration changed with age in a quadratic manner, such that: Gluc = 5.82–0.128×Age + 0.0082×Age^2^ (Fig 1C). Serum Gluc concentration reached its lowest point at 7.8 years. Serum Alb concentration decreased linearly with age by approximately 0.1 g/L per year. Serum TBili concentration decreased linearly with age by approximately 0.014 μmol/L per year.

Age-, but not sex-, related changes were noted in serum total calcium, phosphorus, chloride, and potassium concentrations (Table 1). Serum total Ca concentration decreased nonlinearly with age, such that: Ca = 2.40 + 0.15×*e*^(-0.49×Age)^ (Fig 1A). Total Ca concentration reached the upper limit for the 95% confidence interval (2.392 to 2.403 mmol/L) for the asymptote (2.397 mmol/L) at 6.6 years of age. Similarly, serum Phos concentration decreased nonlinearly with age, such that: Phos = 1.28 + 0.54×*e*^(-1.47×Age)^ (Fig 1B); however, the Phos concentration reached the upper limit for the 95% confidence interval (1.274 to 1.286 mmol/L) for the asymptote (1.280 mmol/L) at 3.1 years of age. Serum Cl concentration increased linearly with age by approximately 0.07 mmol/L per year, and serum K concentration increased linearly with age by approximately 0.01 mmol/L per year.

A weak positive association existed between heart rate and serum Gluc concentration (r_s_ = 0.16, P < 0.0001), as well as between heart rate and WBC count (r_s_ = 0.13, P < 0.0001).

### Sex related changes in hematology and serum biochemistry

#### Hematology

Sex related differences in hematologic analytes and their effect size as a percent in parentheses were identified in Plt (females, 6.0% higher), blood Hgb concentration (females, 0.9% higher), and Hct (females, 0.6% higher; Table 1). There was no difference in WBC count or MCV between females and males (Table 1).

#### Serum biochemistry

Sex related differences in serum biochemical analytes and their effect size as a percent in parentheses were identified in the serum concentration of SUN (females, 14.7% lower), Tbili (females, 12.8% higher), Creat (females, 7.3% lower), Glob (females, 3.8% lower), TP (female, 1.3% lower), Gluc (females, 1.2% higher), Alb (females, 1.0% higher), and Na (females, 0.1% higher). Sex related differences were also identified in the serum activity of ALT (females, 16.6% lower), ALP (females, 4.6% lower), and AST (females, 4.3% lower; Table 1). There was no difference in serum Ca, Phos, Cl, and K concentrations, the urea nitrogen to creatinine ratio, and CK activity between females and males (Table 1).

### Reference intervals

Reference intervals were determined from the last values recorded for 4,804 dogs, comprising 1,868 (38.9%) females and 2,936 (61.1%) males, with no adjustment for age or sex (Table 2). Use of only one data point from an individual, during construction of a reference interval, is recommended by the CLSI guidelines [22].

**Table 2:** Ninety-five percent reference intervals for selected hematologic and serum biochemical analytes for 4,804 dogs being considered for running in the Iditarod Trail Sled Dog Race during a seven-year study period.

Analyte	n	Median	95% Reference interval (study population)	Reference interval (university population)
**Hematologic analytes**				
White blood cells (x10^9^/L)	4802	9.3	5.5–16.6	6.0–17.0
Hematocrit (L/L)	4802	0.49	0.41–0.56	0.35–0.52
Hemoglobin (g/L)	4802	162	137–187	120–180
Mean corpuscular volume (fL)	4800	70.3	65.5–75.5	58.0–76.0
Platelets (x10^9^/L)	4801	303	127–492	200–700
**Serum biochemical analytes**				
Sodium (mmol/L)	4804	145	142–149	141–152
Potassium (mmol/L)	4804	4.5	3.9–5.2	3.9–5.5
Chloride (mmol/L)	4804	112	107–117	107–118
Calcium (mmol/L)	4804	2.4	2.3–2.6	1.9–2.9
Phosphorus (mmol/L)	4803	1.3	0.9–1.7	0.9–1.7
Albumin (g/L)	4804	32	27–37	25–38
Globulin (g/L)	4804	29	24–36	27–44
Total protein (g/L)	4804	61	54–69	51–70
Urea nitrogen (mmol/L)	4804	6.8	3.6–13.9	2.1–10.7
Creatinine (μmol/L)	4804	56.6	38.9–88.4	44.2–132.6
Glucose (mmol/L)	4804	5.5	4.3–6.7	3.8–7.0
Total bilirubin (μmol/L)	4804	2.6	1.7–3.4	1.7–5.1
Alkaline phosphatase (U/L)	4803	34	16–96	7–92
Alanine aminotransferase (U/L)	4803	49	24–145	8–65
Aspartate aminotransferase (U/L)	4803	27	16–61	11–74
Creatine kinase (U/L)	4804	136	68–565	26–310

Reference interval information from a university veterinary hospital is provided for comparison. (Analyzers used for the university reference interval: hematology–Sysmex XN-1000; biochemistry–Beckman Coulter AU680) The university hematology reference intervals were determined based on a correlation study with the lab’s previous hematology analyzer that utilized 20 dogs over one year of age with specific age and sex unknown. The university biochemistry reference intervals were determined from 35 dogs over one year of age with specific age and sex unknown.

The reference intervals are not adjusted for age or sex.

## Discussion

This study represents the largest number of canine blood and serum samples to be analyzed using the same sampling protocol and equipment and is unique in that all dogs had undergone months of extensive endurance training before sampling. As such, our findings of significant effects of age, sex, and the interaction between age and sex on study analytes provides valuable insight into aging in healthy dogs that remain physically active and this information is likely to be transferable to humans. The greatest effects of age were a decrease in serum Ca concentration from 1 to 6.6 years-of-age, a decrease in serum Gluc concentration until 7.8 years-of-age, after which time serum Gluc concentration increased, a decrease in the blood WBC count until 6.9 years-of-age, and a decrease in serum Phos until 3.1 years-of-age. Most of the sex-associated differences in analytes were small and as such were likely to be clinically unimportant; however, five analytes had a difference greater than 5% between the sexes that may warrant sex-specific reference ranges and further investigation. Females had lower SUN (14.7%) and Creat (7.3%) concentrations, lower serum ALT activity (16.6%), and a higher serum Tbili concentration (12.8%) and Plt count (6.0%) than males. Sampling year was found to be significant throughout the study which may be related to slight year-to-year analytical variability in the analyzer performance or differences in the age distribution and proportion of females sampled. The significance of sampling year does not impact our evaluation of the significance of age, sex and the interaction between age and sex as all data were considered simultaneously during statistical analysis.

It should be noted that our study design differed substantially from that of two recent large studies [10, 11] that examined the hematologic (n = 6,046) and serum biochemical (n = 3,045) changes with age and sex in dogs. Both studies analyzed blood samples obtained from dogs admitted to a veterinary referral hospital for diagnostic purposes. A priori, these study populations cannot be assumed to represent healthy dogs. In both cases, the investigators attempted to address potential admission bias by excluding data from dogs with one or more analytes outside the reference interval for their diagnostic laboratory [10, 11]. This approach confounds the characterization of age-related changes as an increase in the number of analytes measured increases the statistical likelihood that at least one parameter from a healthy dog will be outside the reference interval and therefore excluded from analysis. In contrast, we included all data in the analysis based on the assumption that dogs presented for evaluation were considered healthy and fit to race by the musher. This assumption may have led to the inclusion of animals with subclinical disease and is a potential limitation of the study. However, this limitation is present in every reference interval study because resources are never adequate to screen for all subclinical diseases, and because screening tests for subclinical disease rarely have perfect sensitivity and specificity. As such, we believe that acceptable endurance performance, assessed on multiple occasions in the five months before sampling by the musher during training runs, provided a suitable method for assuming a low to negligible incidence of subclinical disease in this dog population.

### Effect of age

Skeletal maturation provided a potential explanation for the decrease in serum total Ca and Phos concentrations from 1.0 to 6.6 and 3.1 years-of-age respectively. Age related decreases in serum total Ca concentration have been documented in the dog, with values appearing to remain stable after 4 years of age [11, 23, 24]. There is a discrepant association between total Ca concentration and age in humans, with a decrease [25], increase [26], or no change [27] with age being reported. Analytes that are physiologically associated with total and ionized Ca concentration, such as serum Phos and Alb concentration [28], were also negatively associated with age in our study population. As the decrease in serum total Ca concentration with increased age was more significant than the decrease in serum Alb concentration with increased age, a decrease in the protein bound fraction of Ca is unlikely to explain all of the change we observed. There is one report of decreased intestinal Ca absorption in older humans [29], most likely due to a decreased ability to transform Vitamin D to 1,25-dihydroxycholecalciferol with increased age secondary to decreased liver function [30] or renal function [31]. Another possible explanation for the decrease in serum Ca in older dogs is an age-related decrease in serum parathyroid hormone (PTH) concentration; however, serum PTH concentrations are increased in older humans [27], with no change in serum total Ca concentration, a decrease in serum ionized Ca concentration, and a decrease in serum Phos concentration with increased age [27]. Because ionized Ca is the biologically active form of Ca in the body, the results of this study suggest that it is important to determine whether ionized Ca concentration also decreases in dogs until 6.6 years-of-age.

The decrease in serum Phos concentration from 1.0 to 3.1 years-of-age is consistent with that observed in other canine studies [11, 24, 32, 33], although increased serum Phos concentrations have been reported in geriatric dogs [12], with the increase assumed to reflect decreased renal function. Decreased serum Phos concentration has been noted in several studies of elderly humans and is thought to be the consequence increased PTH activity [25, 27, 34].

A decrease in serum Gluc concentration from 1.0 to 7.8 years-of-age is a consistent finding in dogs [12, 35, 36], with one report of increased serum Gluc concentration in older dogs [23]. Decreased liver glycogen stores with age have been proposed as the most likely cause for the decrease in Gluc concentration with age in the dog [37], but specific evidence to support this claim is not available. The ability to maintain appropriate serum Gluc concentrations is essential to athletic performance in endurance events, and characterization of the effect of age on muscle glycogen stores would appear to be warranted, as well as determining the effect, if any, of small decreases in serum Gluc concentration on metabolism and endurance performance in sled dogs as well as other sporting dogs. The increase in resting serum Gluc concentration with advanced age in endurance-trained sled dogs is consistent with changes seen in humans [26, 38]. The dog has a long history of being used in type 2 diabetes research as an inducible model of disease [39, 40] and the natural similarities in glucose changes in dogs and humans with age may indicate that the dog should be considered as a model for investigating the effect of exercise on type 2 diabetes in humans. Regular physical activity decreases the risk of type 2 diabetes in humans [41, 42], and former human endurance athletes have the lowest odds ratio for type 2 diabetes with age [42].

Serum Alb concentration was negatively associated with age in dogs in our study and other studies [11, 12, 36], as well as in humans [38, 43]. Causes for decreased serum Alb concentration in older dogs include chronic inflammation, malabsorption/protein losing enteropathy, and protein losing nephropathy. Chronic antigenic stimulation throughout life may lead to longstanding (chronic) inflammation and continuous recruitment of the acute phase response. As albumin is a negative acute phase protein, this continuous recruitment would lead to a decrease in serum Alb concentration over time. This process in the aging individual has been referred to as inflammaging [44]. A low serum Alb concentration is associated with greater loss of appendicular skeletal muscle mass in humans and is consequently a risk factor for sarcopenia [43]. A loss of muscle mass, especially appendicular muscle mass, is likely to negatively impact performance in the canine athlete. The observed weak negative association between age and serum log_10_(CK) activity (r_s_ = - 0.06) or serum log_10_(AST) activity (r_s_ = -0.05) in our study was consistent with decreased muscle mass in older dogs and the findings of another study in dogs [36]. However, no association between serum CK activity and age was identified in a second study in dogs [23], and an increase in serum CK activity in dogs >10 years old was attributed to increased recumbency with age [11]. Findings regarding the association of serum AST activity with age in dogs are inconsistent with no association [23], a positive association [35], and a negative association [36] being reported. The older dogs in our study were considered unlikely to have decreased muscle mass because serum Creat concentration was unchanged, and a positive correlation between Creat and lean body mass has been previously observed in the dog [45]. Further investigation into the relationships between muscle mass, serum Creat concentration, serum CK activity, and age appear indicated to identify the preferred analyte to monitor muscle mass in endurance-trained dogs. Ultrasonographic measurement of the cross-sectional area of selected muscles, such as the longissimus dorsi, might be helpful in this matter [46]. Neuter status information would also be beneficial as neuter status affects serum Creat concentration in male and female dogs and serum CK activity in male dogs [11].

Serum Tbili concentration does not change with age in humans [38, 47]. In the dog, serum Tbili concentration has tended to increase with age, without attaining statistical significance [11, 23]. Although we found a positive association between serum Tbili concentration and age in our study, the association was weak and not considered to be clinically important.

Serum Cl and K concentrations were positively associated with age in this study, whereas no age-related changes were identified in serum Na concentration. A positive association between serum Cl concentration and age has been noted for humans [26] but has not been previously noted in the dog [23]. The most likely reason for the association was the observed negative association between serum Alb concentration and age [48]. A positive association between serum K concentration and age has been noted for dogs [11, 12, 35] and humans [38]. Possible explanations for this association include age-dependent changes in renal excretion due to decreased renal function, and the presence of dehydration or muscle damage. Decreased renal excretion was unlikely as no other evidence of an age-related decrease in renal function was observed. Dehydration was unlikely due to the lack of an increase in SUN, Creat and Na concentration with age. Muscle damage was considered unlikely as a concurrent increase in serum CK activity, rather than the observed decrease, would be expected.

Hematocrit and blood Hgb concentration decreased linearly with age in the dogs in this study. Anemia of aging is common in humans with an anemia incidence of 11% in men and 10% in women over the age of 65 [49]; however, human studies show variable results for changes in Hgb and Hct with age. Studies have shown that men may have a decrease in Hgb and Hct [26, 50], increase in Hct [47] or stable Hgb and Hct [47, 51] after middle age. Hemoglobin and Hct remain stable or may slightly increase in women throughout life [50, 51]. Changes in blood Hgb concentration and Hct with age are variable in canine studies, with reports of no change with age [24, 35], an increase with age [23] and a decrease with age [10, 12, 52, 53]. In those studies that have also considered sex and sex/age interactions, no difference between the sexes or a sex/age interaction was observed [10]. The positive association between MCV and age may be suggestive of mild vitamin B12 deficiency, which is present in 10–15% of elderly humans but anemia secondary to this deficiency only accounts for 1–2% of true anemia in this population [49]. This finding contrasts with previous canine studies that identified a decrease in MCV with age [12, 24].

The WBC count decreased with age in our study until 6.8 years-of-age, consistent with findings in other canine studies [23, 52]. However, the results of one canine study indicated WBC count decreased until 4–6 years of age with a subsequent increase at 6–8 years of age that remained stagnant until 12 years of age after which the WBC count again increased [10]. Results from the same study also indicated that neutrophil counts peaked at 6–8 and >12 years-of-age [10]. Determination of the change in specific leukocyte counts with age in our study would have helped characterize the leukocyte response as immunologic or inflammatory. The decrease in WBC count with age in dogs is consistent with a decline in the function of the immune system with age (immunosenescence) in vertebrates [54].

A positive association between Plt count and age has been consistently identified in canine studies [10, 12, 23] with one Beagle study noting a negative association [52]. The increase in Plt count with age in our study may have resulted from a reactive thrombocytosis in which case this finding may be further evidence of the occurrence of inflammaging in the dog. Certain inflammatory cytokines, mainly interleukin-6, that increase with inflammation have been noted to stimulate thrombopoietin production and induce thrombocytosis [54]. A thrombocytosis secondary to an epinephrine response is unlikely as the response of a dog to handling and new situations should decrease with age, and many dogs in the study were sampled over multiple years making the situation less novel to the older groups.

### Effect of sex

The magnitude of the difference between serum analyte concentrations or activities in female and male dogs in this study was less than 5% for most of the studied analytes, making the difference unlikely to be clinically significant. Nevertheless, sex-specific reference ranges may be needed for SUN, Creat, and Tbili concentrations, as well as serum ALT activity and blood Plt count in endurance-trained sled dogs, based on a greater than 5% difference in the overall mean values for these analytes for female and male dogs.

Females had higher blood Plt counts and Hgb concentration, Hct, and serum Gluc concentration compared to males. Increased blood Plt counts have been previously noted in female dogs [10, 55] and sex-specific reference ranges for Plt counts have been recommended in Beagles [55]. Overall least squares mean serum Gluc concentration was higher in females in our study whereas a previous canine study showed no difference between the sexes for this analyte [36]. The small increase in blood Plt counts and serum Gluc concentration in females could not be attributed to increased sympathetic stimulation as mean heart rates were similar for females and males. Other causes for an increase in Plt count, such as inflammation or iron deficiency, and increases in Gluc concentration, such as an increased cortisol response, were unlikely based on the other analyte differences between females and males. We also noted increases in blood Hgb concentration and Hct in females relative to males. An erythrocyte count was not performed in our study but a corresponding increase in red blood cell count in female relative to male dogs was considered very likely. This contrasts with what is seen in humans [49–51, 56]. As the females and males in our study would have been cared for under the same conditions within each team, dehydration was unlikely to have occurred in only the females across the study.

Serum Na concentration was increased in females by 0.11 mmol/L relative to males in our study. Although this observation has not been previously reported in the dog, the magnitude is very small and unlikely to be physiologically important. An increase in serum Na concentration occurs in women at menopause, with no change in serum Na concentration in men between the 3^rd^ and 8^th^ decade [38].

Serum protein differences were noted between males and females for TP, Alb, and Glob concentration. Serum Alb concentration was slightly increased in female compared to male dogs; this contrasts with what has been seen in humans where serum Alb concentrations are lower in females up to the 6^th^ decade [38]. Serum TP and Glob concentrations were lower in female relative to male dogs in this study. Lower serum TP and Glob concentrations in female dogs have previously been reported [11]; however, other studies failed to identify a difference between the sexes [33, 36]. Neuter status is associated with serum TP and Glob concentration in both male and female dogs [11]. In our study, a significant interaction between sex and age was observed for serum TP and Glob concentrations. The presence of an interaction effect for serum Glob concentration is at odds with a previous report that the number of blood lymphocytes decreases with age in the dog, with no difference noted for sex [57]. In the same study, it was noted that the percentage of circulating B lymphocytes decreases with age regardless of sex; however, this may not be a good indication of overall immunoglobulin production. Other reports concur with our findings [12, 35]. It should be emphasized that serum Glob concentration in our study is a calculated value that includes all globulin fractions, not just immunoglobulin. Nevertheless, the increase in serum Glob concentration in older dogs lends additional support for an increase in chronic inflammation with age and evidence of inflammaging in the dog, typically characterized by an increase in serum γ-Globulin and IgM concentrations [53, 59]. Identification of the specific proteins that increase with age in the dog is needed to improve our understanding of the inflammatory changes that occur with aging. Of additional interest is whether the lower serum Glob concentration in younger females reflects a decreased humoral immune response, such as decreased immunoglobulin titers following vaccination.

Possible indicators of muscle mass, serum Creat concentration, AST activity and ALT activity, were decreased in female dogs in this study, most likely because female dogs have a lower lean body mass than male dogs [45]. Interestingly, serum CK activity was similar for female and male dogs. Serum Creat concentrations are consistently lower in women than men [25, 38, 58], whereas marginally lower values for serum Creat concentration have been noted in female dogs [11]. Several studies have investigated the relationship between muscle mass/body weight and serum Creat concentration in dogs [45, 59] and humans [60]. Although serum Creat concentration is dependent on body weight in humans [60] and lean body mass or muscle mass in dogs [45, 59], the independent effects of body weight and sex on serum Creat concentration does not appear to have been investigated [45, 60]. Due to the retrospective nature of this study, information about body weight or lean body mass of our population was unavailable; however, body weight measurements would have been helpful in explaining the observed sex differences in serum Creat concentration. Information on neuter status would also have been helpful in this study because serum Creat concentration in male and female dogs, and presumably skeletal muscle mass, is affected by neuter status [11]. As serum AST and ALT activity may also provide information regarding muscle health, the possible causes for the decreased activity of these enzymes in females relative to males may be the same as those for serum Creat concentration. However, these enzymes have not previously been evaluated when investigating the relationship between muscle mass/body weight and Creat concentration and instead are more often used to evaluate whether liver injury is present. Decreased serum AST and ALT activities are often seen as clinically insignificant findings unless indications for decreased liver function are concurrently present, which is not the case in this study population. Aspartate aminotransferase activity decreases in women in the 3^rd^ through 5^th^ decades of life but overall there is an increase in AST activity with age [38]. A significant interaction between sex and age was not found for serum AST activity but was identified for ALT activity in our study, with ALT activity increasing in females with age. Alanine aminotransferase activity has been shown to be increased in male Beagles relative to females [36].

Serum urea nitrogen concentration was lower in female dogs, relative to male dogs. This finding, along with a concurrent lower serum Creat concentration in female dogs, could have resulted from a higher glomerular filtration rate in female dogs, but this was considered unlikely. Studies in humans have identified a similar sex difference in SUN [25, 38], but previous studies in dogs have indicated SUN concentration is higher [36] or similar [45] in female dogs, compared to male dogs. Given the magnitude of the difference in SUN in this study between female and male dogs, additional studies into this finding appear warranted.

Alkaline phosphatase activity was lower in female relative to male dogs, similar to findings in humans [38]. It is unlikely that healthy female dogs have lower serum cortisol concentrations relative to healthy male dogs, but this is a possible cause for a decreased ALP activity. Serum Tbili concentrations were higher in female relative to male dogs. This finding is likely of no clinical significance.

### Reference interval

Comparing subpopulation reference intervals within an animal species using large sample cohorts has the potential to reveal genetic or experiential effects of physiological significance that might otherwise be missed. Statistically significant differences in reference intervals by breed for hematology and serum biochemistry have previously been reported in the dog [7, 10, 11] and cat [8]. Breed-associated statistically significant differences do not always equate to clinically significant differences [7], depending on the breed [9] or analyte [8].

Relevant to our study population, statistically significant differences for hematologic and serum biochemical analytes are present in human athletes compared to age matched controls [56]. As the endurance trained sled dog does not represent a unique breed but does represent a unique canine athlete, determination of reference intervals in this population is required to ensure the proper evaluation and care of these animals. The reference intervals for blood Hgb concentration and Hct in our study are higher than those currently used in the clinical pathology lab at the University of Illinois for canine patients (Hgb 120 – 180g/L, Hct 0.35–0.52L/L). The difference in blood Hgb concentration and Hct reference intervals in our study population is just one clinical example of the usefulness of reference interval data. Gastric ulceration is of concern in endurance-trained sled dogs as previous studies have shown that up to 48% of dogs have gastric lesions after race participation [61], and acute blood loss secondary to gastric ulceration can cause death during race participation [62]. Knowledge of this difference is helpful in identifying anemia in endurance trained sled dogs before race participation.

Although our study includes the largest data set of canine hematologic and serum biochemical analytes ever published and the largest data set of healthy athletic dogs, our study is not without limitations. The lack of information regarding neuter status, the absence of dogs beyond the age of 12 years, the unknown effect of our findings on athletic performance and the variability in management of the dogs by team are all limitations. Information regarding neuter status for both sexes would have been valuable as this has been shown to have some effect on serum biochemical values [11] and would allow for better comparison of our findings to the human literature. Variables that have been shown to be influenced by neuter status include serum urea nitrogen and Creat concentration as well as serum CK activity in males and serum Creat and Tbili concentration in females [11]. Information on neuter status should be included in future canine aging studies. Inclusion of all dogs, regardless of the potential for subclinical disease, when determining the reference interval is a potential limitation of the study. The lack of information beyond a total WBC count is also a limitation of the study as this value is the sum of all leukocyte types and may be more heavily influenced by one specific leukocyte type than another. This is an unfortunate result of the retrospective nature of the study. Future studies should address this issue by reviewing blood smears and performing a manual leukocyte differential. Seasonal variation may influence hormone concentrations in the dog which may in turn influence the concentration or activity of some hematologic and serum biochemical parameters. All samples for this study were collected within the same month (February) each year so effects on our specific population are unlikely but our data may not be translatable to other seasons. The provided information is relevant and beneficial when considering that this data provides a reference interval relevant to the time of year when this population of dogs is preparing to race. Finally, samples were not randomly collected from endurance-trained sled dogs from a defined geographic area; rather, samples were collected from a convenience sample of dogs that were being considered for running in the Iditarod Trail Sled Dog Race. While the majority of dogs originated from Alaska, a minority of teams originated from outside Alaska; for example, the 2018 race had 58 teams from Alaska, 4 from the Yukon Territory and 1 from Alberta in Canada, and 1 team each from Norway and Sweden, as well as Montana and Washington in the United States.

Evaluation of the effect that the observed age and sex changes have on performance would be helpful in providing insight into the clinical significance of these changes. Further evaluation of the measured analytes in the dogs that started and finished the race could provide this information, but this is beyond the scope of the current manuscript. Diet, feeding schedule, training and housing conditions are likely to influence hematologic and serum biochemical analytes and would be different between teams. We accounted for this by nesting dogs within a musher’s team in our statistical analysis which allows us to account for variation between separate teams as well as within the same team between sampling years.

## Conclusion

This study adds valuable insight to canine aging research by focusing on elite endurance trained dogs in the four-week period before a long-distance race. We identified evidence of inflammaging, representing chronic inflammation with age, with a decrease in serum Alb concentration, an increase in serum Glob concentration that differed for female and male dogs, and an increase in Plt count with age. Evidence of immunosenescence, characterized by a decrease in WBC with age, and anemia of aging were also identified. Changes observed in this study but not previously reported in the canine aging literature included a decrease in serum total Ca concentration until 6.6 years-of-age. Although we do not have information regarding the change in ionized Ca with age in this population, appropriate plasma ionized Ca concentrations are vital to obtain optimal muscle function in these endurance athletes. Whether similar age-associated decreases in plasma ionized Ca concentration occurs in dogs therefore warrants additional investigation. Further investigation into the age-related changes noted in our study population may be beneficial in providing optimal nutrition to endurance athletes as well as improving our understanding of metabolic changes in the aging canine and human.
